# Extensive Bone Marrow Necrosis: Initial Presentation in Sickle Cell Anemia—A Case Report and Review of the Literature

**DOI:** 10.1155/2017/7185604

**Published:** 2017-06-13

**Authors:** Sameera A. Alsafwani, Abdulwahed Al-Saeed, Rehab Bukhamsin

**Affiliations:** ^1^Qatif Central Hospital (QCH), Qatif, Saudi Arabia; ^2^Dammam Medical Complex (DMC), Dammam, Saudi Arabia; ^3^Dammam Regional Laboratory and Blood Bank (DRL), Dammam, Saudi Arabia

## Abstract

Bone marrow necrosis (BMN) is a rare clinical entity that was first described in an autopsy of a sickle cell disease (SCD) patient and is defined as ill-defined necrotic cells in an amorphous eosinophilic background with preservation of cortical bone. The pathophysiology of BMN is not well known; however, occlusion of the bone marrow microcirculation with subsequent hypoxia and cell injury has been thought to be common underlying features. Malignancy has been identified to be the primary cause in 90% of the cases whereas SCD was found in only 2%. In this report we present an unusual case of SCD with late onset of the disease whose initial presentation was extensive BMN. The patient was not known previously to have SCD, when suddenly she presented with severe cytopenias and marked elevation in serum lactate dehydrogenase (LDH). Bone marrow examination was done to exclude bone marrow infiltration, and BMN with dilated marrow sinuses full of irreversibly sickled cells were the unexpected findings. Patients with a mild SCD phenotype are at high risk of BMN. Thus, a high index of suspicion must be borne in mind, particularly in an area of high SCD prevalence, to recognize and prevent this catastrophic complication.

## 1. Introduction

Bone marrow necrosis (BMN) is rarely encountered in clinical practice. It was first described in an autopsy of a sickle cell disease (SCD) patient by Wade and Stevenson [1]. BMN refers to necrosis of myeloid tissue and medullary stroma in large areas of the haemopoietic bone marrow that results in an amorphous eosinophilic background, ill-defined necrotic cells with preservation of the cortical bone [2]. The bone marrow trephine shows disruption of normal marrow architecture with loss of fat spaces but generally with preservation of the specular architecture [3]. The incidence of BMN varies among different reports, ranging from 0.3 to 37% [3]. Malignancy has been identified to be the primary cause of BMN in more than 90% of the cases [4]. Other nonmalignant causes include hemoglobinopathies, infections, drugs, anorexia nervosa, hemolytic uremic syndrome (HUS), antiphospholipid syndrome, and disseminated intravascular coagulopathy (DIC) [3, 5, 6]. SCD was found to be the primary cause in only 2% of the cases [4]. Although the pathophysiology of BMN is not well defined, occlusion of the bone marrow microcirculation is assumed to be the initiating factor [7]. Occlusion of the microcirculation could be caused by variable factors such as tumor cell emboli, fibrin thrombi, toxic effect of drugs, radiation, bacterial infection, or cytokines [2, 8, 9]. Patients with extensive BMN usually present with fever, bone pain, and fatigue and have pancytopenia with a leucoerythroblastic picture in the peripheral blood film (PBF) and, characteristically, a striking number of nucleated red blood cells (NRBCs) [10]. Elevated serum lactate dehydrogenase (LDH), alanine transferase (ALT), alkaline phosphates (ALP), and uric acid levels are also common features [4]. Examination of bone marrow biopsy is a prerequisite for the accurate diagnosis of BMN. We report an unusual case of a 26-year-old Saudi female whose bone marrow was referred for evaluation due to her clinical presentation with generalized body aches, jaundice, hepatosplenomegaly with anemia, and thrombocytopenia.

## 2. Case Presentation

**Figure 1:**
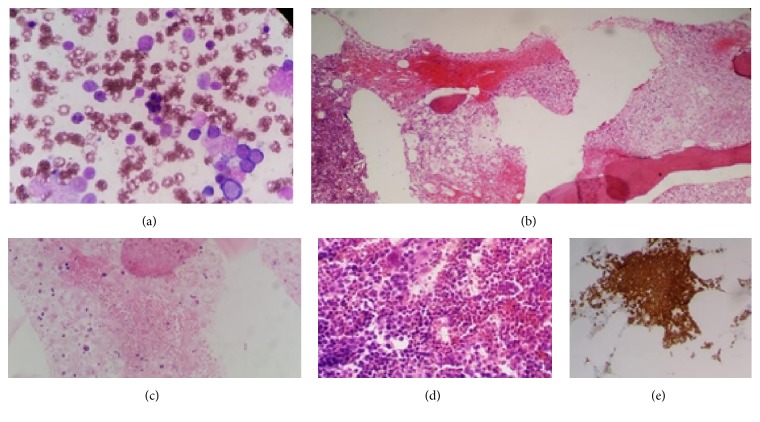
(a) Bone marrow aspirate showing an amorphous necrotic material (Giemsa stain ×100). (b) Extensive BMN in the trephine biopsy (H&E ×4). (c) Dilated sinus full of sickle cells in a background of ill-defined eosinophilic material (H&E ×40). (d) Intact area with erythroid hyperplasia (H&E ×40). (e) Spectrin stain showing the extent of erythroid hyperplasia (IHC, spectrin ×10).

A 26-year-old Saudi female with known diabetes mellitus (DM) type-1 was admitted with jaundice, generalized body aches, and abdominal distention. She gave a history of chest infection that has been treated with antibiotics three days prior to her presentation. The family denied any previous hospitalizations or similar episodes in the past. Her mother and father are second-degree relatives with a history of sickle cell trait (SCT) in the father and DM type-1 in her identical twin sister. On examination the patient was conscious, alert, jaundiced, and not in distress and looked pale. Her vital signs were normal with clear chest examination and had a normal cardiovascular examination. Abdominal examination showed hepatosplenomegaly with ascites and she had intact central nervous system (CNS) examination. The initial blood count showed severe anemia with hemoglobin (Hb) of 5 g/dL (normal range 12.5–18 g/dL) and thrombocytopenia with platelet count of 5 × 10^9^/L (normal range 150–450 × 10^9^/L). Reticulocyte count was 4.2% (normal range 0.5–2.5%). PBF showed polychromasia with a marked leucoerythroblastic picture. Serum LDH was markedly elevated to 3000 U/L (normal range 135–255 U/L) and high total bilirubin 3.1 g/dL (normal range 0–1.2 g/dL), mainly indirect bilirubin. Renal function and other liver enzymes, however, were within the normal range. Computed tomography (CT) scan showed hepatosplenomegaly. Virology screen for corona, H1N1, and parvo virus was negative. During admission, the patient's condition deteriorated with declines in Hb and platelet count and an increase in serum LDH level. The patient was admitted to the intensive care unit (ICU) and bone marrow examination was done to exclude bone marrow infiltration. PBF after transfusion of 10 units of packed red blood cells (PRBC) showed a leucoerythroblastic picture with polychromasia and rare sickle cells. Bone marrow aspirate showed gelatinous basophilic material with distorted morphology. The bone marrow biopsy revealed extensive bone marrow necrosis involving more than half of the one centimeter length biopsy, markedly increased erythropoietic activity in the intact area, and dilated sinusoids which were full of sickled red blood cells (Figure 1). The diagnosis of extensive bone marrow necrosis secondary to sickle cell disease was reported based on these findings. Hb electrophoresis after transfusion showed HbS level of 34%. Family studies were done and revealed that both parents had SCT and her identical twin was found to have SCD with a HbS level of 75%. The patient received supportive therapy and eventually did very well. She subsequently received full vaccinations and began regular follow-up in the hematology clinic. Five months later her Hb electrophoresis showed a HbS level of 80%.

## 3. Discussion

BMN is infrequently encountered in clinical practice. The incidence of BMN varies from 0.3 to 37% among different reports [11]. Such variability in the results could be attributed to the difference in the type of specimens examined (in vivo or postmortem), pathologist experience, and diagnostic criteria used (the incidence was reduced to 0.3 to 12% when only those biopsies with more than half bone marrow involvement by necrosis were included) [11]. BMN is defined as necrosis of hematopoietic tissue and stroma with preservation of cortical bone [2, 4]. It has been identified with various clinical conditions including malignancy, infection, autoimmune disease, chemotherapy, DIC, anorexia nervosa, antiphospholipid syndrome, and sickle cell disease [3–6, 8–10].

Although the first case of BMN was reported in SCD patient, the association of BMN with SCD was reported in only 2% of the cases [4]. One possible cause of the paucity of this association is that bone marrow examination is not commonly done during sickle cell crisis [12]. Charache and Page stated that one of six patients with SCD has some degree of BMN during painful crisis; usually these patients have full recovery [4]. The largest review on BMN in SCD was done by Tsitsikas et al. who identified 58 cases of BMN with fat embolization syndrome (FES) and 16 cases of BMN without FES. In both groups there were a number of patients who were not known to have SCD prior to the presentation of BMN 19 (33%) and 4 (25%), respectively. It has been found that patients with genotype SS were at low risk for BMN/FES and, paradoxically, those with mild phenotypes were at higher risk of this catastrophic complication [10].

SCD is a relatively common genetic disorder in Saudi Arabia with the highest prevalence noticed in eastern province (approximately 21% for SCT and 2.6% for SCD) [13]. There are two major clinical phenotypes of SCD in Saudis. Patients from the western province have the severe phenotype which is consistent with the Benin haplotype. Acute chest syndrome with recurrence, stroke, dactylitis, lower base line total hemoglobin and hemoglobin F level, and early presentation with painful crisis are common clinical features of SCD in the western province. On the other hand patients with SCD from the eastern province have a more benign phenotype which is consistent with the Arab/Indian haplotype. They have a greater incidence of associated deletional alpha thalassemia, higher total hemoglobin and hemoglobin F levels, persistent splenomegaly, more avascular necrosis of the femoral head, and later disease presentation [14].

In our case, the first clinical disease presentation of SCD was extensive bone marrow necrosis with crisis at age of 26 years. She was not suspected to have the SCD prior to her presentation. She had never complained of bone pain and Hb electrophoresis had never been previously performed. Her identical twin sister as well was not known to have the disease nor did she complain of bone pain prior to the family study. Although the presentation of SCD in the eastern province of Saudi Arabia is relatively late as compared with the western province, presentation at this age (26 years old) in our patient and her identical twin is thought to be extremely uncommon. Also, the initial disease presentation with development of extensive BMN is rarely reported in the literature [15–18]. Having a mild SCD phenotype, the limited family history and lack of neonatal or later screening for hemoglobinopathies all contributed to the late diagnosis of SCD and postponed interventions that could possibly prevent life threatening complications of the disease such as BMN. This case presentation emphasizes the importance of SCD screening and need for follow-up of the patients as catastrophic complications could occur irrespective of the disease phenotype. This is especially important in an area with high SCD prevalence such as the eastern province of Saudi Arabia.

Another unique finding in our case is that she has coexistence of SCD and type-1 DM which is rarely reported as well in the literature [19, 20]. Presence of the high prevalence of both diseases in this part of the world could provide a logical explanation for the coexistence of these disorders in these identical twin sisters.

## 4. Conclusions

BMN is infrequently encountered in clinical practice and rarely reported in association with SCD. As the initial presentation of SCD, BMN was previously reported only in a few cases in the literature. Patients with a mild SCD phenotype are at high risk of BMN. Thus, a high index of suspicion must be borne in mind to prevent the development of catastrophic complications particularly, in an area with a high prevalence of SCD.
